# Periodontal Disease and Alzheimer’s: Insights from a Systematic Literature Network Analysis

**DOI:** 10.14283/jpad.2024.79

**Published:** 2024-04-19

**Authors:** Alice Villar, S. Paladini, J. Cossatis

**Affiliations:** 1 Faculty of Medicine, University Castelo Branco (UCB), Rio de Janeiro, Brazil; 2 School of Arts, Social Sciences, and Management, Queen Margaret University, Musselburgh, UK; 3 Faculty of Medicine, University Castelo Branco (UCB), Rio de Janeiro, Brazil

**Keywords:** Alzheimer Disease, periodontal disease, oral health, neurodegenerative diseases, bibliometrics

## Abstract

This study investigated the relationship between periodontal disease (PD) and Alzheimer’s Disease (AD) through a Systematic Literature Network Analysis (SLNA), combining bibliometric analysis with a Systematic Literature Review (SLR). Analyzing 328 documents from 2000 to 2023, we utilized the Bibliometrix R-package for multiple bibliometric analysis. The SLR primarily centered on the 47 most globally cited papers, highlighting influential research. Our study reveals a positive correlation between Periodontal Disease (PD) and Alzheimer’s Disease (AD), grounded in both biological plausibility and a comprehensive review of the literature, yet the exact causal relationship remains a subject of ongoing scientific investigation. We conducted a detailed analysis of the two main pathways by which PD could contribute to brain inflammation: (a) the Inflammatory Cascade, and (b) Microbial Involvement. The results of our SLNA emphasize the importance of oral health in reducing Alzheimer’s risk, suggesting that managing periodontal health could be an integral part of Alzheimer’s prevention and treatment strategies. The insights from this SLNA pave the way for future research and clinical practices, underscoring the necessity of interdisciplinary methods in both the investigation and treatment of neurodegenerative diseases like Alzheimer’s. Furthermore, our study presents a prospective research roadmap to support ongoing advancement in this field.

## Introduction

In recent years, the intersection of oral health and neurodegenerative diseases has become a key focus in scientific research. Among the various neurodegenerative conditions, Alzheimer’s Disease (AD) stands out as one of the most prevalent and devastating, affecting millions of individuals worldwide. While extensive research has shed light on the multifaceted etiology of AD, there remains a growing interest in understanding the potential links between Periodontal Disease (PD), a chronic inflammatory condition of the oral cavity, and the development or progression of Alzheimer’s Disease. This paper presents a comprehensive investigation, utilizing the methodological approach of Systematic Literature Network Analysis (SLNA) to examine the evolving landscape of research at the intersection of PD and AD.

The significance of this study lies in its pursuit of elucidating the connections between periodontal health and cognitive well-being. By systematically analyzing the existing body of literature, we aim to provide valuable insights that may inform future research directions and clinical practices. As a guiding framework for the SLNA, this paper addresses the following research questions (RQs):

- RQ1. What is the nature of the correlation between Periodontal Disease and Alzheimer’s Disease, as identified through this SLNA?
- RQ2. What is the impact of Periodontal Disease on the initiation and advancement of Alzheimer’s Disease?
- RQ3. What is the significance of managing periodontal health in the context of Alzheimer’s prevention and treatment?

Our paper is organized into six sections. In Section 1, we provide an introduction, outlining the research motivation and addressing the identified gap. Section 2 details our methodology, which comprises two phases: Bibliometric Analysis (Section 2.1) and Systematic Literature Review (Section 2.2). Section 3 focuses on our Bibliometric Analysis. Section 4 discusses the findings from our Systematic Literature Review, focusing on insights from the 47 most frequently cited papers identified in the Bibliometric Analysis. Section 5 presents a future research agenda to support further development in the field, and Section 6 concludes the paper by summarizing key insights.

## Methodology - Systematic Literature Network Analysis (SLNA)

**Figure 1:**
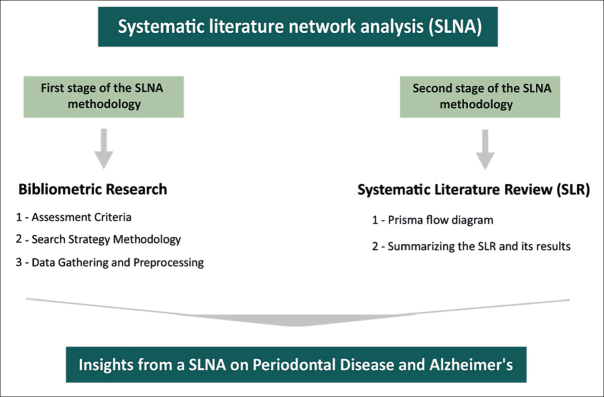
SLNA on Periodontal Disease and Alzheimer’s

In our study, we use SLNA because it combines both qualitative and quantitative approaches, giving us a detailed insight into the topic. This method allows us to see how the research field has evolved over time, spotlighting both well-explored and under-researched areas. The thoroughness of SLNA improves the depth and precision of our investigation. As presented in Figure 1, the execution of our SLNA is divided into two stages: an initial phase of bibliometric analysis, succeeded by a Systematic Literature Review (SLR).

### Methodology for the First Phase of SLNA - Bibliometric Analysis

Our bibliometric analysis methodology is divided into two main phases, as detailed in Table 1. The first phase sets out the criteria for choosing relevant literature, while the second outlines our search strategy. This method led us to identify 328 documents spanning from 2000 to 2023. We utilized the Bibliometrix R-package to analyze these publications and create visual network representations. In Section 3 of our paper, we showcase a series of bibliometric graphs.

**Table 1:** Assessment criteria and Search Strategy Methodology

**PHASE 1: ASSESSMENT CRITERIA**		
**Eligibility criteria**	**Reasoning**	
Timeframe: Published papers from 2000 to 2023	The 2000 to 2023 timeframe was selected to encompass significant developments and trends in the research field over two decades, blending historical depth with recent advancements for a comprehensive and relevant analysis.	
Database Source: Scopus	Scopus database, recognized as the world’s largest abstract and citation database of scientific literature,is more suited for bibliometric research than PubMed, particularly because it includes citation data, essential for our research’s bibliometric analysis.	
Language: English	English remains the predominant language in scientific communication, ensuring our study encompasses the forefront of research developments.	
Chosen search locations within the text: Abstracts	Searching by titles allows for efficient identification of relevant studies, ensuring our query captures the core essence of the research.	
Document type: Article, Review, Conference Paper	The chosen document types, which undergo rigorous peer-review processes, provide high-quality, methodologically sound, and empirically rich content. This diverse range of studies offers robust data and insights for our SLNA.	
**PHASE 2: SEARCH STRATEGY METHODOLOGY**		
**Academic Database**	**Search Query**	**Reasoning**
Scopus	(«periodontal disease» OR «periodontitis» OR «gingivitis» OR « gum disease») AND («Alzheimer’s disease» OR «Alzheimer disease»)	This query searches for documents that mention any form of periodontal disease (like periodontitis, gingivitis, or general gum disease) and Alzheimer’s disease. It looks for resources discussing both gum-related diseases and Alzheimer’s, exploring connections or correlations between them.

### Methodology for the Second Phase of SLNA - Systematic Literature Review (SLR)

**Figure 2:**
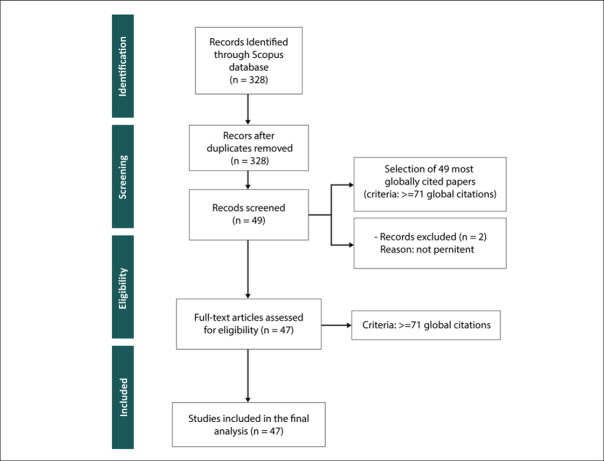
PRISMA flow diagram summarizing the SLR

We conducted a Systematic Literature Review (SLR) using the PRISMA methodology, as illustrated in Figure 2. In the «identification» stage, we incorporated our full collection of 328 documents. These were gathered through our bibliometric analysis conducted using Bibliometrix tool in the first stage of our SLNA Methodology (Figure 1). The «screening» phase involved an evaluation of these documents against predefined criteria, with a focus on the most globally cited papers (≥71 citations). This resulted in the selection of 49 documents. In the «eligibility criteria» stage, 2 documents were not pertinent for inclusion. The «inclusion» stage resulted in a final set of 47 documents for our SLR. This ensured a focused analysis of PD and AD connections.

## Bibliometric Analysis

In Section 3, we showcase our bibliometric analysis, offering a comprehensive overview of the scientific research landscape within our study area.

### Annual Evolution of Scientific Production

**Figure 3:**
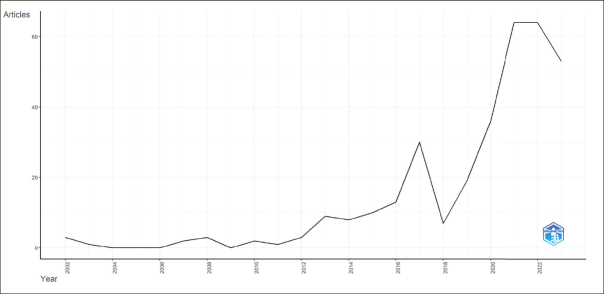
Annual Evolution of Scientific Production

Figure 3 presents the evolution of research connecting PD to AD from 2000 to 2023 based on Scopus data as of October 12, 2023, with the main information about this data detailed in Table 2. It reflects an annual growth rate of 14.65% among the 328 documents.

**Table 2:** Main information about data

**Main information about data**	**Results**
Timespan	2002:2023
Sources (Journals, Books, etc)	184
Documents	328
Annual Growth Rate %	14,65
Document Average Age	4,52
Average citations per doc	39,31
Authors	1519
Authors of single-authored docs	18
Single-authored docs	21
International co-authorships %	33,23

### Wordcloud

**Figure 4:**
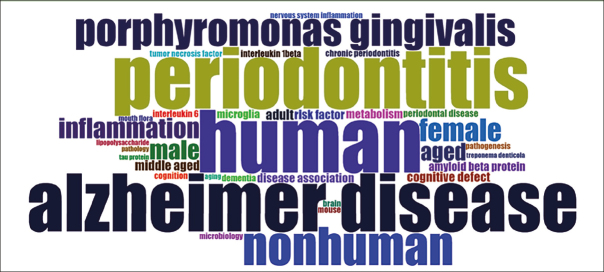
Wordcloud

The wordcloud in Figure 4 was generated from the Keywords Plus parameter of Bibliometrix. It showcases 35 terms that encapsulate the core themes of the literature being reviewed. As a visual representation, the wordcloud displays these terms in varying sizes to reflect their frequency in the dataset; the more often a word appears, the larger and more prominent it is displayed.

In the wordcloud, the term ‘human’ prominently stands out, alongside ‘periodontitis’ and ‘Alzheimer’s disease’, indicating their central role in the scope of the reviewed literature. The considerable representation of ‘nonhuman’ shows the inclusion of comparative studies that extend to animal models. The mention of ‘mouse’ implies the use of laboratory animal studies to dissect disease mechanisms further.

The specific pathogen ‘Porphyromonas gingivalis’ is highlighted, pinpointing a focal area of investigation regarding Periodontal Disease’s impact on neurological health. Gender-related terms and various age groups point towards a demographic analysis within the research, discussing the impact of Periodontal Disease (PD) and Alzheimer’s across different genders and age categories.

Inflammatory markers such as tumor necrosis factor (TNF) and interleukin 6 (IL-6) show a focus on the physiological processes and molecular pathways involved in the relationship between periodontal health and neurodegenerative conditions. Terms such as ‘inflammation’, ‘amyloid beta protein’, and ‘microglia’ indicate key areas of interest in the pathophysiology of Alzheimer’s disease. Additionally, terms like ‘metabolism’, ‘pathogenesis’, and ‘pathology’ indicate a broader scientific interest in the systemic and disease development aspects of the research.

### Keywords Co-occurrence Network (KCN)

**Figure 5:**
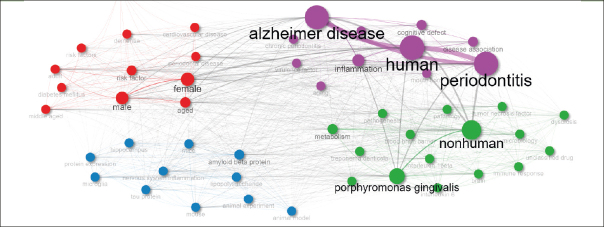
Keywords Co-occurrence Network (KCN)

Figure 5 features a Keywords Co-occurrence Network (KCN) with three clusters, each highlighting a different research theme in Periodontal Disease (PD) and Alzheimer’s Disease (AD).

The Purple Cluster focuses on the human-centered aspects of PD and AD. It includes keywords like ‘human’, ‘periodontitis’, ‘Alzheimer disease’, and ‘inflammation’, emphasizing the direct impact and clinical manifestations of these diseases in humans. The presence of terms such as ‘cognitive defect’, ‘chronic periodontitis’, ‘cognition’, and ‘aging’ indicates a focus on how PD and AD affect cognitive functions and the aging process. Terms like ‘mouth flora’ and ‘virulence factor’ show an exploration into the role of oral microbiota and their contribution to disease progression.

The Blue Cluster delves into the biological and molecular mechanisms linking PD and AD. It comprises terms like ‘amyloid beta protein’, ‘microglia’, ‘tau protein’, and ‘lipopolysaccharide’, highlighting research on the pathological agents and processes involved in these diseases. Terms like ‘mouse’, ‘mice’, and ‘animal experiment’ points towards the utilization of animal models to study disease mechanisms. This cluster also explores neurological aspects with terms like ‘hippocampus’ and ‘protein expression’, indicating an investigation into how these diseases affect the brain at the molecular level.

The Red Cluster addresses demographic and risk factors associated with PD and AD. It includes terms like ‘female’, ‘male’, ‘aged’, ‘adult’, and ‘middle aged’, emphasizing the study of these diseases across different genders and age groups. This cluster also incorporates ‘risk factor’, ‘periodontal disease’, ‘dementia’, ‘diabetes mellitus’, and ‘cardiovascular disease’, highlighting the exploration of various risk factors and comorbid conditions that influence the prevalence and progression of PD and AD. The term ‘pregnancy’ in this cluster shows a specific interest in understanding how these diseases affect pregnancy outcomes.

The Green Cluster addresses broader biological and systemic aspects of PD and AD. It encompasses terms such as ‘nonhuman’, ‘porphyromonas gingivalis’, and ‘metabolism’, indicating a focus on the systemic and microbiological factors. ‘Interleukin 1beta’, ‘interleukin 6’, ‘tumor necrosis factor’, and ‘immune response’ highlight the immunological response involved in these diseases. The inclusion of ‘pathology’, ‘dysbiosis’, ‘genetics’, and ‘blood brain barrier’ suggests a comprehensive exploration of the genetic, microbiological, and systemic impacts of PD and AD.

### Most Global Cited Documents

**Figure 6:**
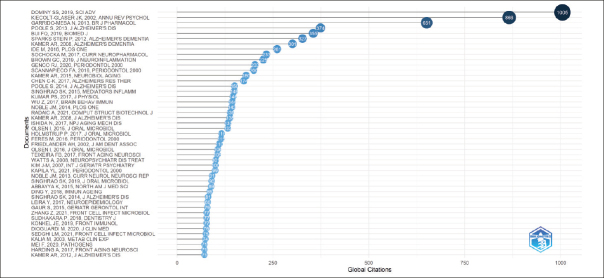
Most Globally Cited Documents

Figure 6 showcases the 47 Most Globally Cited Documents. Each document in this selection has been chosen for having a minimum of 71 citations. Global Citations means the Total Citations that an article, included in the collection, has received from documents indexed on a bibliographic database (WoS, Scopus, etc.). So, TC counts citations received by a selected article «all over the world» (5). Choosing to focus on global citations in our SLR is pivotal as it directs our focus towards the most impactful and recognized publications within our field of study.

In our systematic literature review (SLR), while we initially concentrated on the 47 top-cited documents globally, our research findings extend beyond these sources. To deepen our literature review, we engaged in further qualitative research through platforms like PubMed and Google Scholar. By performing targeted searches using keywords similar to those in Table 1, we were able to embrace a broader spectrum of perspectives and include a richer variety of academic contributions.

## Systematic Literature Review

Our Systematic Literature Review (SLR) starts with a detailed examination of the pathogenesis of Alzheimer’s Disease (AD) and Periodontal Disease (PD) in Section 4.1, laying the groundwork for understanding these conditions. Next, in Section 4.2, we explore the neuroinflammatory pathways that connect PD and AD, uncovering the key mechanisms involved.

Our Systematic Literature Review (SLR) advances with a structured analysis segmented into three distinct categories: human studies, animal studies, and review articles. The section on animal studies is presented in Table 3 (Section 4.3), followed by the human studies, which are detailed in Table 4 (Section 4.4). The review concludes with Section 4.5, summarizing the findings from review articles listed in Table 5. This final section offers a broad overview of the current state of research and points out areas where further investigation is needed.

**Table 3:** Animal Studies: Periodontal Disease and Alzheimer’s Connection

**Nº**	**Author and Year**	**Study Objectives**	**Population**	**Key Biomarkers**	**Animal Model**	**Results**	**Type of Study**
1	POOLE S et al. (2015)	Examine the invasion of oral pathogens into the brain and its effects on complement activationin ApoE-/- mice.	Mice with oral P. gingivalis infection	Gingipains, Aβ1–42, neuroinflammation markers	Mice	Gingipain inhibitors reduced P. gingivalis load and neurotoxicity in the brain.	Animal Experimental Study
2	WU Z et al. (2017)	Assess the role of cathepsin B in Alzheimer’s disease-like phenotypes following exposure to lipopolysaccharide from Porphyromonas gingivalis in mice.	Wild-type and Cathepsin B-deficient mice exposed to P. gingivalis LPS	Cathepsin B, IL-1β, TLR2, amyloid precursor protein (APP)	Wild-type and CatB-deficient mice	Cathepsin B plays a critical role in neuroinflammation and neural dysfunction in response to P. gingivalisLPS.	Animal Experimental Study
3	DING Y et al. (2018)	Explore the effects of Porphyromonas gingivalis infection on memory impairment and neuroinflammation in mice.	Young and middle-aged C57BL/6 J mice infected with P. gingivalis	Pro-inflammatory cytokines (TNF-α, IL-6, IL-1β)	C57BL/6 J mice	P. gingivalis infection caused cognitive impairment and increased proinflammatory cytokines in middle-aged mice.	Animal Experimental Study
4	DOMINY SS et al. (2019)	Investigate the presence of Porphyromonas gingivalis in Alzheimer’s disease brainsand assess the effects of gingipain inhibitors.	Mice with oral P. gingivalis infection	Gingipains, Aβ1-42, neuroinflammation markers	Mice	Gingipain inhibitors reduced P. gingivalis load and neurotoxicity in the brain.	Animal Experimental Study
5	SINGHRAO SK et al. (2019)	Evaluate the role of Porphyromonas gingivalis in periodontitis and its potential causal relationship with Alzheimer’s disease in mouse models.	Various mouse models with Porphyromonas gingivalis or LPS introduction	Amyloid-beta plaques, phosphorylated tau, neurofibrillary tangles	Various mouse models	P. gingivalis and LPS introduction resulted in neuropathological lesions and memory impairment.	Animal Experimental Study
6	BROWN GC et al. (2019)	Test the endotoxin hypothesis of neurodegeneration using rodent models to explore the role of lipopolysaccharide.	Rodents exposed to high levels of endotoxin	Amyloid β, tau, TDP-43, microglial activation markers	Rodents	Endotoxin induced neurodegeneration, amyloid β and tau aggregation in rodents.	Animal Experimental Study

**Table 4:** Human Studies: Periodontal Disease and Alzheimer’s Link

**Nº**	**Author and Year**	**Study Objectives**	**Population**	**Key Biomarkers**	**Results**	**Type of Study**
1	KIECOLT-GLASER JK et al. (2002)	Explore the relationship between negative emotions and various diseases influenced by the immune system.	General human population with various health threats.	Proinflammatory cytokines.	Negative emotions can intensify health threats, potentially through distress-related immune dysregulation.	Review/Conceptual
2	SPARKS STEIN P et al. (2012)	Examine serum antibody levels to periodontal bacteria in Alzheimer’s disease patients compared to control subjects.	158 participants in the BRAINS research program.	IgG antibodies to oral bacteria.	Elevated antibodies to F. nucleatum and P. intermedia were found in AD patients compared to controls.	Cohort Study
3	POOLE S et al. (2013)	Establish a link between periodontal disease and Alzheimer’s disease by identifying major periodontal bacteria in brain tissue postmortem.	10 Alzheimer’s Disease cases and 10 non-AD controls postmortem.	Lipopolysaccharide fromP. gingivalis.	LPS from periodontal bacteria was found in AD brain tissue, suggesting an inflammatory role in AD pathology.	Human Postmortem Study
4	KAMER AR et al. (2015)	Examine the association between periodontal disease and brain amyloid load in elderly individuals.	38 cognitively normal, healthy, elderly individuals.	Brain amyloid load (11C-Pittsburgh compound B PET imaging).	Clinical attachment loss was associated with increased brain amyloid load in vulnerable regions.	Human Postmortem Study
5	IDE M et al. (2016)	Investigate the association between periodontitis and cognitive decline in Alzheimer’s Disease.	60 community dwelling participants with mild to moderate Alzheimer’s Disease.	Serum pro-inflammatory cytokines.	Periodontitis is associated with increased cognitive decline in Alzheimer’s Disease and increased systemic inflammation.	Observational Cohort Study
6	CHEN C-K et al. (2017)	Determine whether chronic periodontitis increases the risk of developing Alzheimer’s disease.	9291 patients with chronic periodontitis and 18672 matched controls.	Not specified.	10-year exposure to chronic periodontitis was associated with a 1.707-fold increase in the risk of developing Alzheimer’s disease.	Retrospective Matched-Cohort Study

**Table 5:** Review Articles on Periodontal Disease and Alzheimer’s Association

**Nº**	**Author and Year**	**Study Objectives**	**Results**	**Type of Study**
1	KAMER AR et al. (2008a)	Explore the link between periodontitis and Alzheimer’s disease risk.	Periodontitis may play a role in the onset and progression of Alzheimer’s disease.	Review
2	WATTS A et al. (2008)	Exploring the role of inflammation in linking periodontal disease to AD.	Inflammation may mediate the association; gene polymorphisms may influence this relationship.	Review
3	KAMER AR et al. (2008b)	Explore chronic periodontitis and inflammation’s role in Alzheimer’s disease.	Chronic periodontitis, a treatable peripheral infection, may affect AD’s development and progression.	Review
4	GARRIDO-MESA N et al.(2013)	To explore the pharmacology of minocycline and its therapeutic applications beyond its antibiotic properties.	Oral inflammation may contribute to a range of systemic diseases.	Review
5	SINGHRAO SK et al.(2014)	Examining the link between periodontitis and AD progression.	Periodontal pathogens may contribute to AD progression.	Review
6	SINGHRAO SK et al. (2015)	To explore the potential connection between periodontal pathogen P. gingivalis and AD.	Suggests a possible contribution of P. gingivalis to AD etiology.	Review
7	ABBAYYA K et al. (2015)	To review the link between periodontitis and AD.	Inflammation serves as a link between periodontitis and AD.	Review
8	SCANNAPIECO FA et al. (2016)	To assess the impact of oral diseases onsystemic conditions in the elderly.	Oral diseases may influence systemic conditions; improved oral hygiene might prevent aspiration pneumonia.	Review
9	HARDING A et al. (2017)	To explore the relationship between periodontitis, AD, and nutritional factors.	Links between chronic periodontitis, nutritional aspects, and AD; highlights potential for diet-based interventions.	Review
10	SOCHOCKA M et al.(2017)	Investigating the potential causative role of infections in the progression of AD.	Infections, especially neurotropic viruses and certain bacteria, might contribute to the inflammatory pathway in AD.	Review
11	KUMAR PS et al. (2017)	To review the role of the oral microbiome in systemic disease.	Periodontal pathobionts may play a role in systemic diseases such as cardiovascular disease, rheumatoid arthritis, Alzheimer’s disease, and others.	Review
12	SUDHAKARA P et al.(2018)	To explore the impact of oral dysbiosis on systemic diseases.	Oral dysbiosis is linked to systemic diseases such as cardiovascular disease,diabetes, rheumatoid arthritis, and Alzheimer’s disease.	Review
13	BUI FQ et al. (2019)	To investigate the link between periodontitis and various systemic diseases.	Indications of a link between periodontitis and diseases like cardiovascular disease, diabetes, Alzheimer’s disease.	Review
14	KONKEL JE et al. (2019)	Discussing the systemic effects of oral inflammation.	Oral inflammation may contribute to a range of systemic diseases.	Review
15	GENCO RJ et al. (2020)	To understand the impact of periodontitis on systemic health.	Links with chronic noncommunicable diseases, including diabetes, cardiovascular disease, metabolic diseases, rheumatoid arthritis, cancers, respiratory diseases, and cognitive disorders.	Review
16	DIOGUARDI M et al. (2020)	To review the connection between periodontitis, its bacteria, and AD.	Periodontal bacteria may intensify inflammation contributing to AD.	Review
17	MEI F et al. (2020)	Assessing the impact of P. gingivalis on systemic diseases.	P. gingivalis linked to atherosclerosis, Alzheimer’s disease, rheumatoid arthritis, and other diseases.	Review
18	ZHANG Z et al. (2021)	Investigating the role of P. gingivalis OMVs in periodontal and systemic diseases.	P. gingivalis OMVs contribute to systemic impacts of periodontal disease.	Review
19	SEDGHI LM et al. (2021)	Synthesizing knowledge on periodontal disease and its systemic impacts.	Link between periodontal disease and systemic conditions like cardiovascular diseases and Alzheimer’s disease.	Review
20	RADAIC A et al. (2021)	To explore the oral microbiome’s role in health and disease management strategies.	Dysbiotic oral microbiome can lead to systemic diseases including cardiovascular disease, diabetes, Alzheimer’s disease, and cancer.	Review

### Pathogenesis of Alzheimer’s and Periodontal Disease

Alzheimer’s disease (AD) is a progressive neurodegenerative disorder mainly defined by three primary pathological characteristics:

(a) Aβ (Amyloid-beta) Plaque Accumulation: In AD, there is an excessive accumulation of amyloid-beta (Aβ) plaques in the brain, especially in regions like the cerebral cortex and hippocampus. While Aβ plays beneficial roles under normal conditions, its overproduction or inadequate clearance results in the formation of toxic plaques. These plaques are known to contribute to neuronal damage (33).

(b) Tau Hyperphosphorylation: In AD, the tau protein, which plays a crucial role in stabilizing microtubules in neurons, undergoes abnormal and excessive phosphorylation. This excessive phosphorylation prevents tau from properly attaching to the microtubules. As a result, the microtubules within the axons of neurons become destabilized, leading to the creation of neurofibrillary tangles (NFTs). These tangles contribute to the loss of synaptic connections and a decline in cognitive function.

(c) Microglia-Mediated Neuroinflammation: In AD, the brain’s immune cells, known as microglia, become chronically activated. This prolonged activation causes persistent neuroinflammation. The activated microglia produce pro-inflammatory cytokines and substances harmful to neurons, worsening neuronal damage and accelerating the disease’s progression (64).

Akiyama et al. (2) underscore the critical role of inflammation in the development of AD and suggest that focusing on inflammatory processes could pave the way for anti-inflammatory treatments to potentially slow down or delay the onset of AD. While research on anti-inflammatory medications, particularly Non-Steroidal Anti-Inflammatory Drugs (NSAIDs), appears promising for AD management, their actual effectiveness is still a matter of debate. Observational studies indicate potential benefits, yet these findings have not been consistently replicated in clinical trials. Given the current lack of conclusive clinical evidence, NSAIDs are not recommended for individuals experiencing cognitive decline or those diagnosed with AD, as noted by Asthana et al. (4) and Ali et al. (3).

Periodontal Disease (PD), scientifically termed periodontitis, is chronic infection caused by multiple types of bacteria. This condition is primarily characterized by the proliferation of a specialized ecosystem known as dental biofilm, which adheres to the tooth surface. The predominant periodontal pathogens involved in periodontitis are Aggregatibacter actinomycetemcomitans (Aa), Porphyromonas gingivalis (Pg), Prevotella intermedia (Pi), Fusobacterium nucleatum (Fn), Tannerella forsythensis (Tf), Eikenella corrodens (Ec), and Treponema denticola (Td). (15)

The dental biofilm contains a diverse population of about 400 bacterial species (48). Beyond bacteria, it also includes substances produced by these bacteria, like endotoxins and virulence factors, as well as a mixture of protein-containing and non-protein substances. This composite structure creates a favorable environment for the growth and survival of periodontal bacteria, while also protecting them from the body’s immune system and antibiotic treatments. The initial phase of periodontitis is marked by the establishment of plaque biofilms, which are heterogeneous conglomerates of multiple microbial species. These biofilms act as a catalyst for an inflammatory response within the periodontal region. Persistent inflammation, if left unchecked, can progressively inflict damage upon pivotal periodontal structures, including the periodontal ligaments and the alveolar bone. Without proper treatment, this degenerative process can lead to the loosening or even loss of teeth (46, 64).

In periodontitis, dysbiosis, or an imbalance of oral bacteria, triggers increased inflammation, especially in those with genetic susceptibility. This leads to the breakdown of periodontal components like alveolar bone, ligaments, and cementum. Importantly, shifts in the composition of oral bacteria typically occur before the clinical signs of periodontitis become evident. Certain bacteria found subgingivally are associated with more severe forms of the disease. A prime example is Porphyromonas gingivalis (P. gingivalis), a gramnegative bacterium, characterized by the presence of lipopolysaccharides (LPS) and gingipains as part of its structure. These substances are pivotal in causing tissue damage, exacerbating inflammation, and altering immune responses during the disease’s progression.

Dominy (14) further underscores the significance of P. gingivalis in the context of Alzheimer’s disease, revealing its presence in the brains of AD patients. The study found that gingipains, toxic proteases from P. gingivalis, correlate with tau and ubiquitin pathology in Alzheimer’s patients, indicating a link to neurodegeneration. Additionally, oral P. gingivalis infection in mice showed brain colonization, increased production of amyloid-beta (Aβ1–42), and neurotoxic effects on tau. This research led to the development of small-molecule inhibitors targeting gingipains, which showed promise in reducing P. gingivalis brain infection, blocking Aβ1–42 production, and alleviating neuroinflammation in Alzheimer’s disease.

The body responds to periodontitis by producing inflammatory cytokines like TNF-α, IL-1β, and IL-6, which play crucial roles in the disease’s progression. TNF-α and IL-1β stimulate cells that degrade bone and periodontal tissues. Meanwhile, IL-6 not only accelerates bone loss but also promotes the conversion of B cells into plasma cells, which are involved in the immune response. The high levels of IL-6 found in patients with periodontitis highlight its important role in the disease’s development (54, 64).

Numerous studies have found that bacteria associated with Periodontal Disease (PD) can infiltrate gum tissues and enter the bloodstream during routine activities like chewing and tooth brushing, or during dental procedures, potentially causing temporary, recurrent bloodstream infections (40). These periodontal areas act as ongoing sources for the spread of bacteria and inflammation-causing agents throughout the body. This phenomenon, as described by Hajishengallis (20), demonstrates that periodontitis is not only a local oral health issue but also a dysbiotic inflammatory disease with significant systemic implications. Hajishengallis emphasizes that the dysbiosis in oral microbial communities associated with periodontitis can mediate inflammatory pathology not just locally in the oral cavity but also at distant sites in the body, increasing the risk for various systemic conditions.

When these bacteria and inflammatory substances reach distant organs, they may trigger similar inflammatory reactions, leading to organ-specific diseases (46). Chronic PD has been linked to neurodegenerative disorders like Alzheimer’s and a range of other conditions, including cardiovascular disease (62, 6), oral cancer (35, 26), colorectal cancer (11, 39), rheumatoid arthritis (50), gastrointestinal issues (66, 17), respiratory infections (44, 18), adverse pregnancy outcomes (49), diabetes (63, 28), insulin resistance (61).

### Neuroinflammatory Pathways Linking Periodontal Disease to Alzheimer’s

Chronic inflammation from Periodontal Disease (PD) is increasingly recognized as a potential risk factor for Alzheimer’s disease (AD), with implications for increased disease risk and progression (42, 58, 7, 34).

In the 2018 case-control study led by Holmer J. et al. (23), they found that individuals with mild cognitive impairment (MCI), subjective cognitive decline (SCD), and AD had a higher prevalence of poor oral health indicators compared to controls. The odds ratios for factors such as marginal alveolar bone loss and deep periodontal pockets ranged from 3.36 to 8.43. These findings suggest a possible link between marginal periodontitis, a type of gum disease, and the early stages of cognitive impairment leading to AD.

Martande et al. (41) found that individuals with AD experience more severe periodontal damage, worsening as the disease progresses, emphasizing the need for enhanced oral healthcare in AD patients. This observation underscores the need for heightened oral healthcare for those with AD. It’s crucial for both individuals with Alzheimer’s and their caregivers to be well-informed about maintaining good oral health. Martande et al. noted a strong link between declining periodontal health and cognitive impairment in AD patients. In this same line, recent studies indicate a bi-directional relationship: individuals with dementia are more prone to developing PD, with those experiencing early-onset dementia often facing more severe PD (22, 64).

**Figure 7:**
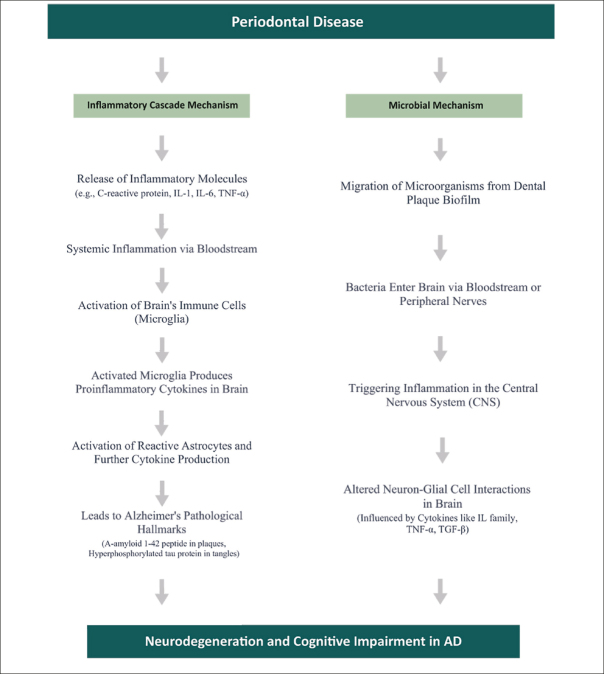
Periodontal Disease to Alzheimer’s: Neuroinflammatory Pathways

The precise pathways linking PD to AD are not yet fully understood and still being explored. However, two main pathways by which PD could contribute to brain inflammation have emerged: (a) the Inflammatory Cascade, and (b) Microbial Involvement (9, 30, 19). Figure 7 presents a summary of the two main pathways by which Periodontal Disease (PD) could contribute to brain inflammation.

#### The Inflammatory Cascade mechanism

The Inflammatory Cascade mechanism is a critical concept in understanding the intersection of Alzheimer’s Disease (AD) and Periodontal Disease (PD). This mechanism suggests that inflammatory molecules originating from PD can intensify brain inflammation, influencing the progression of AD.

Building on this understanding, recent studies have increasingly focused on the role of inflammation in the development and progression of AD. Researchers, including Kamer et al. (29), propose an inflammatory hypothesis for AD. This hypothesis suggests that the persistent oral inflammation associated with PD, perpetuated by itself, exacerbates the neurodegenerative processes typical in AD. While direct inflammatory causes for AD are yet to be conclusively established, it’s theorized that the pathological hallmarks of AD — such as A-amyloid 1–42 peptide (A42) in senile plaques and hyperphosphorylated tau protein (P-Tau) in neurofibrillary tangles — could act as triggers for inflammation.

Periodontal disease (PD) contributes to systemic inflammation by releasing mediators such as C-reactive protein (CRP), interleukins (IL-1, IL-6), tumor necrosis factor (TNF)-α, and α-1-antichymotrypsin into the body’s circulation. These substances, known precursors of cognitive decline, activate the brain’s immune cells, microglia, thus heightening immune response. These pathological changes prompt glial cells to produce proinflammatory brain cytokines. These cytokines can further activate primed glial cells, potentially resulting in an amplified reaction and the progression of conditions like AD.

In cases of chronic periodontitis, increased levels of inflammatory mediators cause microglia to become more responsive to inflammatory signals. This heightened responsiveness could accelerate neurodegeneration in AD, as evidenced in studies by Kamer et al. (32) and Holmes et al. (2009). Although chronic periodontitis might not directly cause AD, it is believed to accelerate its progression, especially in genetically predisposed individuals.

Delving deeper, Kamer (29) notes the connection between senile plaques in AD and reactive astrocytes and activated microglia. These cells respond to inflammatory mediators like TNF-α, IL-1, and IL-6, potentially driving further production of A42, P-Tau, and other proinflammatory molecules, thereby reinforcing a cycle of ongoing inflammation and neurodegeneration.

Microglia react to agents like lipopolysaccharides (LPS) by kickstarting the central nervous system’s innate immune response. This activation releases neurotoxic elements such as cytokines, complement factors, and reactive oxygen species, exacerbating inflammation-linked pathologies (46). Periodontopathic microorganisms and the host response amplify systemic inflammation, which can breach the blood-brain barrier (BBB), leading to microglial activation and potential neuronal damage (1). In this process, known as the Inflammatory Cascade, periodontal pathogens and the body’s response increase levels of inflammatory cytokines, leading to widespread inflammation. These cytokines can compromise the BBB, invading brain areas and possibly causing neuronal damage (19).

Additionally, research shows a correlation between elevated antibodies to periodontal bacteria and AD, which might assist in its clinical diagnosis. Notably, Kamer et al. (2009) found a significant correlation between the presence of elevated antibodies against periodontal bacteria and the incidence of AD. This research revealed that patients with AD demonstrated higher plasma levels of Tumor Necrosis Factor-alpha (TNF-α) as well as an increased number of antibodies targeting periodontal bacteria, compared to normal, healthy controls. These findings suggest that both elevated TNF-α and the heightened immune response to periodontal pathogens could serve as potential biomarkers for the clinical diagnosis of AD.

#### The Microbial Mechanism

The Microbial Mechanism focuses on the diverse microbiome of the oral cavity. Emerging studies suggest that bacteria associated with Periodontal Disease (PD) could enter the bloodstream and subsequently reach the brain. This migration poses a potential risk of triggering or exacerbating the pathology of Alzheimer’s Disease (AD).

A crucial aspect of this mechanism is the movement of microorganisms from the dental plaque biofilm to the brain. This transfer can happen either via the bloodstream or along nerve pathways. Studies conducted by Kamer et al. (30) indicate that once these microorganisms or their by-products arrive in the brain, they could induce inflammation within the central nervous system (CNS). Such inflammation is a critical element in the cognitive deterioration seen in AD. The cognitive decline in AD is largely due to detrimental interactions between neurons and glial cells, fueled by inflammatory cytokines. Key cytokines in this process include the interleukin (IL) family, tumor necrosis factor-alpha (TNF-α), transforming growth factor-beta (TGF-β), and various chemokines. These play a crucial role in the pathogenesis of AD and are crucial for early detection, acting as biomarkers to signal the disease’s onset. TNF-α, in particular, is known for its role in exacerbating inflammation, leading to gliosis, demyelination, blood-brain barrier breakdown, and cell death, thereby playing a central role in neurodegeneration (30, 29, 1, 19).

Studies using mouse models have shown the beneficial effects of anti-inflammatory agents in reducing neuroinflammation and amyloid plaque formation. These agents also significantly decrease levels of IL-1β and glial fibrillary acidic protein in treated mice, highlighting their potential in managing AD-related inflammation (1).

### Animal Studies on the Association between Periodontal Disease and Alzheimer’s

This section examines findings from animal studies on the link between Periodontal Disease (PD) and Alzheimer Disease (AD), primarily based on papers in Table 3 («Animal Studies: Periodontal Disease and Alzheimer’s Connection») and includes insights from additional relevant research.

Several studies suggest a link between periodontitis and AD, but the causal mechanism remains unclear. Wu Z et al. (65) investigated this by examining the role of cathepsin B (CatB) in AD-like symptoms induced by chronic exposure to Porphyromonas gingivalis (P. gingivalis) lipopolysaccharide (PgLPS) in mice. Their findings reveal that PgLPS exposure leads to learning deficits and amyloid-beta accumulation in middle-aged mice, with increased CatB expression associated with neuroinflammation. This research suggests CatB as a potential therapeutic target for preventing cognitive decline associated with periodontitis in Alzheimer’s disease.

Dominy et al. (14) found P. gingivalis, a gum disease bacterium, and its toxic gingipains in Alzheimer’s patients’ brains, associated with key Alzheimer’s markers. In mice, oral P. gingivalis infection led to brain invasion, increased amyloid plaque component Aβ1–42 (a component of amyloid plaques), and tau protein damage. The study highlighted that gingipain inhibitors can reduce brain infection, lower Aβ1–42, reduce neuroinflammation, and protect neurons, suggesting their potential in treating Alzheimer’s linked to P. gingivalis.

The study by Poole S et al. (47) demonstrated that the oral pathogen P. gingivalis can invade the brains of ApoE-/- mice, with its genomic DNA detected in 50% (6/12) of the mice brains at 12 weeks (p = 0.006) and 75% (9/12) at 24 weeks post-infection (p = 0.0001). Additionally, there was significant complement activation observed in the hippocampal neurons, as evidenced by opsonization with C3 activation fragments in 33% (4/12) of the infected mice brains (p = 0.032). These results suggest that periodontal disease may contribute to Alzheimer’s disease by allowing oral pathogens to invade the brain, triggering an immune response that could lead to neuronal damage.

Ding Y et al. (13) found that middle-aged mice infected with P. gingivalis, a periodontitis-causing bacterium, showed impaired learning and memory and increased brain levels of pro-inflammatory cytokines TNF-α, IL-6, and IL-1β. This suggests that P. gingivalis infection may lead to cognitive impairment through neuroinflammation in an age-dependent manner.

Ilievski, V et al. (25) showed that oral exposure to P. gingivalis in mice led to AD-like features: neuroinflammation, neurodegeneration, amyloid beta plaques, and neurofibrillary tangles. Pg/gingipain was found in the brain, particularly in microglia, astrocytes, and neurons. Increased pro-inflammatory cytokines (IL6, TNFα, IL1β), neurodegeneration markers, amyloid-related genes (APP, BACE1), extracellular Aβ42, and phosphorylated tau protein supported the link between periodontal pathogen exposure and AD-like neuropathology.

The study by Singhrao et al. (56) explores the link between chronic periodontitis and Alzheimer’s disease (AD), suggesting that a long-term periodontitis of 10 years may double the risk of developing AD. Their research showed that P. gingivalis infection or its lipopolysaccharide (LPS) in mice models leads to AD’s key neuropathological features and cognitive impairments.

Brown GC et al. (8) explored the endotoxin hypothesis of neurodegeneration, positing that endotoxin, a component of gram-negative bacteria, contributes to neurodegenerative diseases. They found that endotoxins can induce inflammation and activate brain microglia, potentially exacerbating neurodegeneration. While the hypothesis is still unproven, their study indicates that reducing endotoxin-induced neuroinflammation could be a therapeutic strategy.

Ishida N. et al. (27) found that inducing periodontitis with P. gingivalis in transgenic mice worsened Alzheimer’s disease (AD) features. The mice with periodontitis had impaired cognitive function and increased amyloid-beta (Aβ) deposition in the brain, along with elevated pro-inflammatory cytokines (IL-1β and TNF-α). This suggests that periodontitis may exacerbate AD by promoting brain inflammation and Aβ accumulation.

### Human Studies on the Relationship between Periodontal Disease and Alzheimer’s

In this section, we analyze various human studies that explore the link between Periodontal Disease (PD) and Alzheimer Disease (AD). Our primary references are the papers listed in Table 4. Furthermore, we incorporate insights from other relevant studies to enrich our understanding of this association.

Chen et al. (10), in a retrospective cohort study using Taiwan’s National Health Insurance Research Database, found that patients with Chronic Periodontitis (CP) for 10 years had a significantly increased risk of developing AD compared to those without CP (adjusted hazard ratio [HR] 1.707, p=0.0077). This suggests a strong link between long-term periodontal disease and AD risk.

Sparks Stein et al. (58) examined serum antibody levels to periodontal disease bacteria in participants who later developed AD compared to control subjects. It found significantly higher antibody levels to Fusobacterium nucleatum and Prevotella intermedia in patients who developed AD, with statistical significance set at a = 0.05. These findings, which were consistent even after adjusting for age, cognitive status, and genetic factors, suggest a potential link between PD and an increased risk of AD.

Poole et al. (46) investigated the presence of major periodontal disease bacteria and their components in the brains of AD patients. They demonstrate that lipopolysaccharide (LPS) from P. gingivalis, a key periodontal pathogen, is present in the brain tissues of AD patients. This was observed in four out of ten AD cases, with a statistically significant association (p = 0.029). This suggests that LPS from oral pathogens may reach the brain and play a role in the inflammation observed in AD pathology.

Kamer et al. (33) expanded on these findings by showing a significant correlation between PD and increased amyloid β (Aβ) plaque accumulation in the brain (p=0.002), which is a hallmark of AD. This was determined using 11C-PIB PET imaging and adjusting for factors like age, ApoE genotype, and smoking. These results align with previous animal studies, linking peripheral inflammation or infections to brain Aβ accumulations.

Singhrao SK. et al. (55) focused on the role of periodontal pathogens like P. gingivalis in causing inflammation that may affect the brain. They discuss how these bacteria, through everyday activities like brushing or flossing, can enter the bloodstream and potentially reach the brain, contributing to the development of AD in susceptible individuals. Their paper emphasizes the role of chronic infection and inflammation from PD in causing neuron loss and memory deterioration, potentially exacerbating AD.

Ide et al. (24) explored the relationship between periodontitis and cognitive decline in Alzheimer’s disease, focusing on the impact of periodontitis on dementia severity and systemic inflammation. They found that periodontitis was not related to the initial cognitive state in Alzheimer’s patients but was associated with a six-fold increase in cognitive decline over six months, as measured by the ADAS-cog. Additionally, periodontitis was linked to an increase in systemic pro-inflammatory markers, suggesting that the cognitive decline might be mediated by effects on systemic inflammation.

Additionally, Kiecolt-Glaser et al. (36) highlights the connection between negative emotions and various common diseases among older adults, including Alzheimer’s and periodontal disease, through their impact on the immune system. It explains that negative emotions can stimulate the production of proinflammatory cytokines, which are linked to these diseases. It concludes that managing negative emotions might lead to better health outcomes by positively affecting the immune and endocrine systems.

The significance of oral health is further reinforced by the link between tooth loss and cognitive decline. Stein et al. (57) found that having fewer teeth was linked to a higher risk of developing dementia, indicating that poor oral health may be a risk factor for dementia onset. Similarly, Okamoto et al. (45) found that tooth loss in the elderly significantly increases the risk of developing mild memory impairment (MMI), with each additional lost tooth raising the odds by a ratio of 1.02. Being completely toothless (edentulous) was associated with a 2.39 times higher risk of MMI compared to having a full set of teeth. This highlights tooth loss as a potential risk factor for memory decline in the elderly.

### Synthesis of Review Articles on Periodontal Disease and Alzheimer’s

In synthesizing various review articles on the link between Periodontal Disease (PD) and Alzheimer Disease (AD), we begin by examining foundational studies like those of Kamer et al. (29) and Kamer et al. (30), which propose a link between periodontitis and Alzheimer’s based on central nervous system inflammation. These studies highlight the potential of treating periodontitis as a modifiable risk factor for Alzheimer’s disease. This notion is echoed in the works of Watts et al. (60) and Abbayya et al. (1), who explore the inflammatory mediators as potential links between PD and AD.

Further insights are provided by Singhrao et al. (55, 56), who explore how periodontal pathogens, particularly Porphyromonas gingivalis, contribute to Alzheimer’s by causing chronic inflammation in the central nervous system. Similarly, Dioguardi et al. (12) investigate the link between PD and AD, suggesting that periodontal bacteria and related inflammation may impact Alzheimer’s by affecting central nervous system inflammation.

Garrido-Mesa et al. (2013) discuss the role of minocycline, a medication with non-antibiotic effects, in treating periodontitis and its potential neuroprotective effects in Alzheimer’s disease. In addition, research by Watts et al. (60) explores the role of infectious pathogens and inflammation in AD, suggesting that managing PD could offer new strategies for preventing and treating AD.

The studies conducted by Scannapieco et al. (52) and Harding et al. (21) both discuss the relationship between periodontitis and systemic health, including the progression of Alzheimer’s disease. They emphasize the importance of oral hygiene in the context of systemic inflammation and AD. Scannapieco et al. focus on the broader implications of oral diseases in the elderly and the role of oral hygiene in preventing systemic complications. Meanwhile, Harding et al. delve into the specifics of how chronic periodontitis, particularly when caused by pathogens like Porphyromonas gingivalis, can lead to systemic inflammation and the progression of Alzheimer’s disease, also highlighting the influence of diet and microbial imbalances.

Harding et al. (21) explore dietary considerations, highlighting the advantages of adhering to a diet that is calorically balanced, low in carbohydrates, and contains sufficient amounts of protein and fat. This nutritional approach is presented as an effective method to prevent and/or slow down the progression of both periodontal disease and Alzheimer’s Disease.

The concept of «focal infection theory» is brought into focus by Kumar et al. (38), which postulates that oral microbiome infections may cause various chronic diseases, including AD. Sudhakara et al. (59) examine the impact of oral dysbiotic communities on systemic diseases, including Alzheimer’s. They emphasize how deviations from a balanced oral microbiome, termed «dysbiosis,» involving keystone pathogens, can lead to systemic conditions like periodontitis, which are linked to various systemic diseases.

Genco et al. (16) provide an overview of the clinical and public health implications of periodontitis, particularly its association with systemic diseases including AD. Their work underscores the increasing body of research that establishes links between periodontitis and various systemic conditions, such as metabolic diseases, rheumatoid arthritis, specific cancers, respiratory issues, and cognitive disorders like Alzheimer’s.

Bui et al. (9) investigate the connection between periodontitis and systemic diseases, including Alzheimer’s, exploring how pathogens in periodontal disease can influence conditions beyond the oral cavity. Konkel et al. (37) further elaborate on this by reviewing the extensive impact of periodontitis on various chronic diseases, with a specific focus on its association with cognitive decline.

Sedghi et al. (53) and Mei et al. (43) provide insights into the broader impacts of periodontal disease. Sedghi et al. discuss the role of oral pathogens and environmental factors in periodontal disease, linking it to systemic conditions like inflammatory bowel diseases, cardiovascular diseases, Alzheimer’s disease, and COVID-19. Mei et al. specifically focus on Porphyromonas gingivalis and its association with systemic diseases such as atherosclerosis, AD, rheumatoid arthritis, and diabetes. They also mention its potential role in other conditions.

Zhang et al. (67) investigate the impact of Porphyromonas gingivalis (P. gingivalis) outer membrane vesicles (OMVs) on periodontal disease and systemic health, including their potential role in diseases like AD. The study highlights how these OMVs, carrying virulent factors, contribute more significantly to disease pathogenesis than the bacteria itself, emphasizing the importance of oral health in managing systemic diseases.

Radaic et al. (51) review the concept of the ‘oralome’, focusing on the dynamic interactions between the oral microbiome and the host, and how these interactions shift from healthy to dysbiotic states. They discuss the implications of oral dysbiosis, particularly in relation to periodontitis and its association with systemic diseases, including Alzheimer’s disease, outlining potential strategies for managing dysbiosis to prevent and treat these conditions.

## Discussion

Our results corroborate previous studies that have highlighted the role of two main pathways for the Periodontal Disease (PD) – Alzheimer Disease (AD) link: the Inflammatory Cascade mechanism and Microbial Involvement. The Inflammatory Cascade suggests that inflammatory molecules from PD can intensify brain inflammation, a key factor in Alzheimer’s Disease. Microbial Involvement indicates that bacteria from PD might reach the brain and contribute to AD pathology. Importantly, our study enhances comprehension of the PD and AD connection through an in-depth analysis. We offer a simplified flow diagram for better accessibility across different audiences, crucial for this interdisciplinary topic.

Our research identifies a bi-directional correlation between PD and AD (RQ1). On one hand, the chronic inflammation and bacterial load associated with PD might contribute to the development or exacerbation of Alzheimer’s Disease. On the other hand, cognitive decline in AD might lead to deteriorating oral health, further exacerbating PD. Our findings (RQ2) indicate that PD impacts both the onset and progression of AD. The inflammatory cascade and microbial involvement in PD suggest how oral health impacts brain neurodegeneration. This is particularly critical for those genetically predisposed to AD, where ongoing oral inflammation and periodontal pathogens may accelerate AD progression, especially in early stages of cognitive impairment. The systemic impact of PD, including chronic inflammation and immune responses, extends to overall brain health, potentially affecting AD’s key pathological features, like amyloid-beta processing and neurotoxic inflammation.

The correlation identified between PD and AD underscores the importance of periodontal health management as an integral part of a comprehensive strategy for AD prevention and treatment. This connection suggests that maintaining good oral health could play a key role in mitigating the risk and progression of AD. This finding is particularly relevant for healthcare providers, highlighting the importance of integrating dental care with routine medical check-ups for individuals at risk of or diagnosed with AD. This connection warrants further research into how periodontal treatments could influence neurodegenerative processes in AD and the potential role of dental professionals in multidisciplinary teams managing AD. In educating patients, especially those in high-risk groups, about the link between oral health and brain health, healthcare professionals can encourage more proactive and preventive care strategies.

Building upon our findings, our study proposes the following key Research Questions (RQs) for further investigation:

- Epidemiological and Longitudinal Studies: How do the impacts of PD on AD evolve over time, and what are the geographical and environmental factors influencing their co-occurrence?
- Genetic and Molecular Mechanisms: What genetic and molecular biomarkers link PD and AD, providing a more direct connection between these conditions?
- Biomarkers and Neuroimaging: Can combining neuroimaging with oral health biomarkers enhance early-stage AD prediction accuracy?
- Microbiome and Immune System Studies: How do the oral and gut microbiome, and immune responses to periodontal pathogens, influence PD and AD development?
- Public Health and Prevention Strategies: How can public health policies and preventive strategies be shaped to address both PD and AD, considering lifestyle and environmental risk factors?
- Technological Innovations and Digital Health: How can AI and digital health tools predict AD risk based on PD severity and optimize early detection and management of both conditions?
- Clinical Approaches and Therapeutic Interventions: What are the effects of different periodontal treatments on AD’s cognitive functions, and what new interventions can target the PD-AD interface?

These questions guide the future scope of research, emphasizing the importance of periodontal health in AD prevention and treatment. They also highlight the need for comprehensive studies to establish a clearer causative relationship between PD and AD and explore new therapeutic strategies. As we advance, it’s crucial to delve deeper into these areas, enhancing our understanding and ability to combat these interconnected health challenges effectively.

In our investigation into the connection between PD and AD, there are important limitations to consider. Firstly, our primary data source was the Scopus database. While Scopus provided a strong foundation for our analysis, it may not have included all relevant studies, especially those not listed within its database. To address this, we broadened our research to include qualitative studies from other sources, such as PubMed and Google Scholar, enhancing the overall comprehensiveness of our work. Another notable limitation of our study is the substantial reliance on animal-based research, particularly studies involving mice and rats. This emphasis on animal models highlights the need for future research to focus more on human subjects, in order to deepen our understanding of the topic.

Despite these limitations, our study highlights crucial areas for future research focused on enhancing our understanding of the link between PD and AD. It underscores the importance of developing practical strategies that address neurodegenerative diseases through better oral health care. Emphasizing the need for a holistic approach in managing chronic conditions like Alzheimer’s, our findings add significant value to existing knowledge and lay the groundwork for future research directions.

## Conclusion

In conclusion, our Systematic Literature Network Analysis (SLNA) of 328 documents reinforced the association between Periodontal Disease (PD) and Alzheimer’s Disease (AD) and provided an examination of the two primary pathways underlying this relationship. By highlighting the inflammatory cascade and microbial involvement, our study sheds light on biological pathways that could be targeted for future research.

Our findings underscore the systemic nature of PD and its potential role in exacerbating neurodegenerative processes in AD. This recognition calls for a paradigm shift in how we approach the management of PD—not merely as an oral health issue but as a significant factor in overall brain health and neurodegenerative disease prevention.

Looking forward, our analysis highlights several key areas for future research. These include the need for longitudinal studies to better understand the progression of PD and its impact on AD, the exploration of genetic and molecular biomarkers linking these conditions, and the development of targeted therapeutic interventions. Additionally, our study advocates for the integration of oral health into broader preventive and treatment strategies for AD, emphasizing the importance of interdisciplinary approaches in healthcare.

## Data Availability

*Availability of data and material:* In our commitment to research transparency and reproducibility, we have documented our work and methodologies in a publicly accessible GitHub repository. *Code availability:* https://github.com/alicevillar/SLNA_PD-AD.

## Notes

*Ethics approval:* Not applicable.

## Notes

*Consent to participate:* Not applicable.

## Notes

*Consent for publication:* Not applicable.
